# Strategies for Selecting Routes through Real-World Environments: Relative Topography, Initial Route Straightness, and Cardinal Direction

**DOI:** 10.1371/journal.pone.0124404

**Published:** 2015-05-20

**Authors:** Tad T. Brunyé, Zachary A. Collier, Julie Cantelon, Amanda Holmes, Matthew D. Wood, Igor Linkov, Holly A. Taylor

**Affiliations:** 1 U.S. Army Natick Soldier Research, Development and Engineering Center, Natick, Massachusetts, United States of America; 2 Tufts University, Department of Psychology, Medford, Massachusetts, United States of America; 3 U.S. Army Engineer Research and Development Center, Vicksburg, Mississippi, United States of America; University of Muenster, GERMANY

## Abstract

Previous research has demonstrated that route planners use several reliable strategies for selecting between alternate routes. Strategies include selecting straight rather than winding routes leaving an origin, selecting generally south- rather than north-going routes, and selecting routes that avoid traversal of complex topography. The contribution of this paper is characterizing the relative influence and potential interactions of these strategies. We also examine whether individual differences would predict any strategy reliance. Results showed evidence for independent and additive influences of all three strategies, with a strong influence of topography and initial segment straightness, and relatively weak influence of cardinal direction. Additively, routes were also disproportionately selected when they traversed relatively flat regions, had relatively straight initial segments, and went generally south rather than north. Two individual differences, extraversion and sense of direction, predicted the extent of some effects. Under real-world conditions navigators indeed consider a route’s initial straightness, cardinal direction, and topography, but these cues differ in relative influence and vary in their application across individuals.

## Introduction

Traditional explanations of route planning behavior suggest that people rely primarily on relative path geometries and select a route that maximizes utility while maintaining efficiency [1–3]. For instance, a traveler selects a route because it affords visits to particular goal locations while also minimizing route length and complexity. However, emphasizing only utility and efficiency fails to adequately capture inherent peculiarities of human wayfinding behavior. For instance, when choosing between routes, humans tend to select long and straight initial route segments, select the last available turn when approaching a destination, and tend to avoid north-going routes [4–9]. Critically, these strategies are sometimes applied at the cost of selecting a relatively suboptimal route. Thus, while humans indeed employ rational strategies such as utility and efficiency maximization, they also employ strategies that reliably bias route selection away from normative predictions [10]. The present study considers two specific instantiations of this: the initial segment strategy, and the southern route preference, which traditionally have been considered only in isolation. The initial segment strategy and southern route preference describe the tendencies to select paths with relatively straight initial segments, and heading toward the south rather than north, respectively. We investigate how these two strategies might interact in shaping route selection, particularly when routes traverse real-world topographical variations that carry inherent traversal costs [11].

## Related Research

### Route Planning Heuristics

Planning routes from an origin to destination involves identifying a range of route options that afford efficiency while maintaining utility. The process of narrowing down route options for selection involves the application of several path selection criteria [12]. In ranked order these include path length, path efficiency (considering complexity, density, traffic), and utilitarian preferences (e.g., scenery, visiting a particular location). Furthermore, evidence for several less intuitive criteria has emerged. Brunyé and colleagues find that when participants plan routes around cities (Pittsburgh, PA, and Chicago, IL, USA) they tend to prefer routes that go generally south rather than north [9], suggesting that route planners monitor cardinal directions when planning routes. The apparent preference for route planners to select south- rather than north-going routes is termed the *Southern Route Preference*. Follow-on research suggests that the southern preference may be driven by implicit associations between cardinal direction and elevated topography (i.e., north/south is higher/lower elevation, respectively), and that such an association is reliable across diverse topographies characterizing international regions such as Sofia, Bulgaria, Enschede, Netherlands, and Padua, Italy [4].

An additional heuristic was identified by Bailenson and colleagues, who found that when participants chose between equal-length routes they tended to select one with a relatively straight path emanating from the origin [7,8], termed the *Initial Segment Strategy*. Follow-up research suggests that this strategy is likely due to both a minimization of curves and turns, and also the extent to which the initial segment heads in the global direction of a destination [6].

Though the Southern Route Preference and Initial Segment Strategy have been examined in isolation, their relative influence and potential interactivity remain unknown. When two route options exist a route planner would be expected to show strong preferences toward selecting the route that heads generally to the south rather than north, and/or has a straight initial path segment that emanates in the global direction of a destination. If one route holds both of these properties then the probability of selecting that route would presumably increase, as opposed to when only one or neither property is a feature of a route, though no research has examined such a possibility. This may be especially true when route options are of equal length, introducing inherent ambiguity into the decision-making process; of course, a relatively lengthy route is unlikely to be selected even when it is initially straight and heads south (as suggested by ranked criteria; [12]).

### Wayfinding Theory

Why might individuals adopt such strategies during route planning? Wayfinders use an incremental process to select successive path segments along a route, and routes are not typically fully planned in advance [13]. This property of route planning might promote a focus on initial aspects of a route such as the straightness and cardinal direction of the first path segment. Focusing on initial path segments and easily extracted geometric properties (e.g., straightness, cardinal direction) likely reflects effort toward cognitive economy when making choices under uncertainty. In other words, when there is no rationally correct route option (i.e., they are identical lengths) wayfinders employ heuristics, or rules of thumb, that simplify an otherwise complex problem [14]. This involves activating (often implicitly) learned adaptations that offer simple principles for arriving at cognitively efficient [15] solutions, such as weighting reliance on initial paths emanating from an origin, and instantiating associations between topography and cardinal direction. This type of spatial decision making has been termed a *least-decision-load* strategy that minimizes the quantity of information that needs to be processed and maintained regarding route options [16]. In many cases, this process may result in selecting optimal routes. In other cases, an overreliance on relatively superficial and spatially constrained path features may result in suboptimal route selection. Identifying additive influences of multiple initial path features, such as straightness and initial cardinal direction, would support theories positing cognitive economy and least-decision-load strategies.

In explaining wayfinding behavior, theories tend to focus on distinctions between attention paid to the low-level properties and features of paths (e.g., turns, landmarks, curves, width), versus attention paid to the global direction of a path (e.g., the angular difference between a position and a goal) [13,17]. To our knowledge, no existing research has considered how topography fits into such a model, though it likely proves particularly important for wayfinding through large-scale space. In small-scale environments, such as a few city blocks or a suburban neighborhood, routes are unlikely to traverse topographically complex areas. Indeed most routes are traversed in restricted scale and highly familiar environments, such as commuting to and from an employment location, or walking around your neighborhood [11]. Perhaps for these reasons extant research tends to neglect or deemphasize understanding how relative topography along a route might influence path selection [12,18]. In large-scale environments, such as through valleys and mountainous region, relative topography likely plays a larger role in influencing route selection. For instance, when given two equal-length route options—one remaining in a valley and the other temporarily traversing a mountainous region—the cost associated with traveling through mountains may prove a particularly powerful influence on route selection. Assuming no known utility (e.g., visiting a particular location) for entering the mountainous region, relatively flat terrain affords faster travel while consuming fewer metabolic (i.e., during locomotion) or economic (i.e., gasoline during driving) resources [11,19]. The influence of relative topography on route selection, however, remains unknown. Furthermore, it is unclear how topography might interact with application of the southern route preference and initial segment strategy. If relative topography indeed influences path selection, the present data will empirically validate Golledge’s [12] initial hypothesis, and advance our understanding of how large-scale global properties of a path guide decisions. It will also begin to highlight map features, including both paths and relatively natural features that influence spatial decision making.

### Individual Differences

Another factor that may influence route planning behavior is individual differences in skills, preferences, and traits. Efforts toward modeling and predicting human wayfinding behavior in transportation contexts have benefitted when including factors related to individual skills and propensities [20,21]. To continue exploring the potential utility of individual differences in predicting spatial behavior, the present study includes a battery of individual differences measures related to spatial skills, spatial preferences, and relatively domain-general decision making styles and personality traits. Though these were included on an exploratory basis, we made some basic hypotheses. First, individuals with higher spatial skills may consider a broader range of route characteristics and thus show lower overall application of the southern route preference and initial segment strategy. Second, individuals with more aversion toward uncertainty (i.e., more inclination to be decisive), or lower openness, may rely more upon heuristics in guiding route selection to establish concrete strategies and quickly reach decisions [22]. Such findings would better inform theoretical models of route selection, and aid modeling efforts that attempt to predict route selection for applications such as locating isolated personnel, recovering the lost, and traffic management [23–25].

### The Present Study

Earlier research has demonstrated that when wayfinders select between two equal-length routes, their choices can be partially predicted by the extent to which a route’s initial segment heads toward a goal’s global direction, and the extent to which it heads to the south rather than north [4,6–9]. There is also some suggestion that topography plays an important, but unclear, role in route planning [11]. No research to date, however, has assessed the potential interactivity or relative influence of these three factors.

To address these issues, the present study used satellite imagery and a route planning scenario similar to that used in prior work [8,9], and used a factorial repeated-measures design to manipulate the cardinal direction (north, south, east, west) of routes, the initial straightness of routes (straight, winding), and the relative topography of regions traversed by possible routes (flat-flat, mountainous-mountainous, flat-mountainous). Participants selected one of two routes overlaid on real-world satellite imagery, and we measured the relative frequency of selected routes. We also administered questionnaires probing for individual differences in spatial skills, spatial strategies and preferences, and decision-making styles.

We made four primary hypotheses. First, we expected to replicate past work and find evidence for both the southern route preference and initial segment strategy. Second, we expected that topographic features would predict route selection, with participants disproportionately selecting routes traversing relatively flat versus elevated terrain. Third, we expected additive influences of initial segment straightness, cardinal direction, and topography. Furthermore, we expected that relative topography, given its direct influence on energy expenditure and traversal time, would exert the strongest influence over route selection. Finally, we expected that individual differences in spatial skills, spatial strategies and preferences, and decision making styles and personality traits may modulate the relative influence of initial segment straightness, cardinal direction, and topography.

## Method

### Ethical Compliance

Research was approved by the Tufts University Social, Behavioral, and Educational Research Institutional Review Board (SBER IRB; protocol #1310028), with secondary approvals from the funding agency (U.S. Army Human Research Protections Office). Participants provided written informed consent to participate in the study.

### Participants & Design

One hundred and four individuals participated for monetary compensation; data from four participants were removed due to failure to correctly perform the task. The resulting sample consisted of 73 females and 27 males (*M*_age_ = 20.4). Each participant completed a series of questionnaires and then viewed a set of 368 images; on each image, the participant selected a single route from two options connecting a depicted origin and destination. In a within-participants design, route options varied systematically in their overall cardinal direction of travel (north, south, east, west), their initial straightness (straight, winding), and the type of topography traversed (flat, mountainous).

### Materials

We developed 320 experimental and 48 filler images that varied in relative topography, cardinal direction, and initial straightness of routes. Images were sourced from Google satellite images (scale on screen: 3cm = 3.2km). To avoid the possibility that our U.S. participants would recognize sampled regions, we sourced exclusively from the Asian continent (China, Thailand, Indonesia, Cambodia, North Korea, South Korea, Vietnam, Laos, Burma).

#### Experimental images & routes

To develop the experimental images we conformed to a 10 (Topography) × 8 (Route Direction) × 4 (Initial Segment Features) factorial design (see Table 1), resulting in 320 images. We began by identifying regions conforming to three topographical characteristics: all flat, all mountainous, and mixed flat and mountainous (approximately equal proportions). In total, we identified 32 flat regions, 32 mountainous regions, and 128 mixed flat and mountainous regions. The mixed regions varied between four conditions that divided the environment vertically, horizontally, or diagonally (32 each): North mountains & South flat, South mountains & North flat, Northwest mountains & Southeast flat, and Southeast mountains & Northwest flat. Each of these 128 mixed flat and mountainous regions was rotated 90° clockwise to create the other 128 mixed combinations: East mountains & West flat, West mountains & East flat, Northeast mountains & Southwest flat, and Southwest mountains & Northeast flat (respectively). Each of the 320 resulting images measured 1200×1200 pixels.

**Table 1:** Experimental factors, with 10 levels of Topography, 8 levels of the Route Direction, and 4 levels of Initial Segment Features.

Experimental Factors		
Topography (10)	Route Direction (8)	Initial Segment Features (4)
FLAT	NORTH→SOUTH	BOTH STRAIGHT
MOUNTAINOUS	SOUTH→NORTH	BOTH WINDING
NORTH MOUNTAINS & SOUTH FLAT	EAST→WEST	LEFT STRAIGHT & RIGHT WINDING
SOUTH MOUNTAINS & NORTH FLAT	WEST→EAST	LEFT WINDING & RIGHT STRAIGHT
EAST MOUNTAINS & WEST FLAT	NORTHWEST→SOUTHEAST	
WEST MOUNTAINS & EAST FLAT	NORTHEAST→SOUTHWEST	
NORTHEAST MOUNTAINS, SOUTHWEST FLAT	SOUTHEAST→NORTHWEST	
NORTHWEST MOUNTAINS, SOUTHEAST FLAT	SOUTHWEST→NORTHEAST	
SOUTHEAST MOUNTAINS, NORTHWEST FLAT		
SOUTHWEST MOUNTAINS, NORTHEAST FLAT		

To confirm that participants could readily differentiate mountainous versus flat satellite image regions, we conducted a small pilot study (N = 8). Results of this pilot study demonstrated near perfect accuracy (overall *M* = .998, *SD* = .005) when participants were asked to click on the mountainous region of the 256 mixed flat and mountainous images.

Two routes were overlaid on each image, both emanating from a single origin (indicated by marker icon A) to a single destination (indicated by marker icon B). Origin and destination marker icons were arranged north/south along the vertical axis, east/west along the horizontal axis, and southwest/northeast and southeast/northwest along the diagonal. Routes connecting the origin and destination markers were overlaid onto the images using Adobe Illustrator, and the two routes depicted on a single image were always the same pixel length. When possible, our plotted routes followed actual routes depicted on the original satellite images, though we note that this occurred in a vast minority of cases. Across images we balanced the travel direction of the route (e.g., going from north to south or vice-versa). Depicted routes never provided a direct-line connection between the origin and destination. In east/west conditions the routes traveled generally north or south before arriving at the destination; in north/south conditions the routes traveled generally east or west before arriving at the destination; in diagonal conditions, routes traveled generally away from and then back to the diagonal before arriving at the destination.

**Fig 1:**
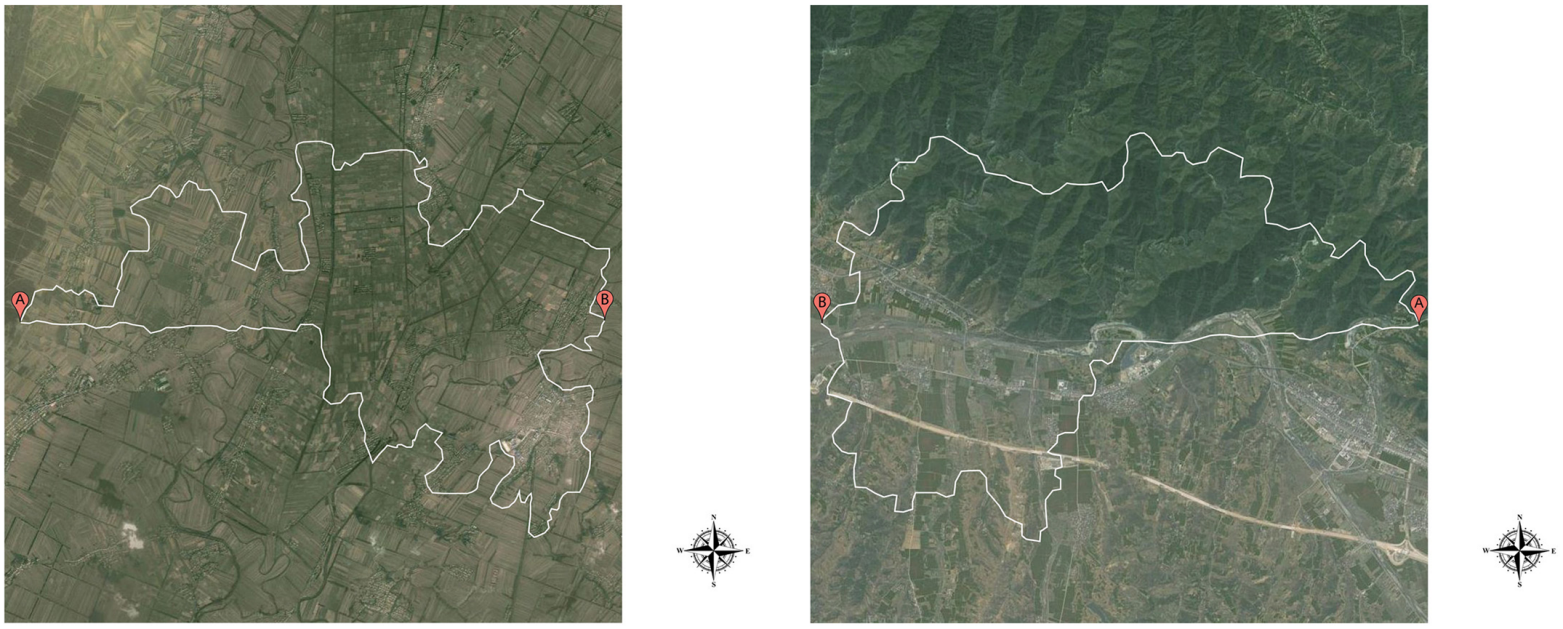
Sample route options. Fig 1a depicts routes going West to East overlaid on flat topography; the southern option depicts a straight initial segment, and the northern option a winding initial segment. Fig 1b depicts routes going East to West overlaid on mixed (North mountainous, South flat) topography; the southern option depicts a straight initial segment, and the northern option a winding initial segment.

To manipulate initial segment straightness we varied route straightness from the origin across four conditions, described here from the perspective of standing at the origin and facing the destination: both straight, both winding, left straight and right winding, and right straight and left winding. The initial segment always emanated in the global direction of the destination marker and extended in a relatively straight (fewer curves/turns) line for approximately 50% of the straight-line distance between the origin and destination. Fig 1 depicts sample images conforming to these constraints.

#### Filler images & routes

We developed 48 filler images in an effort to make the manipulation less apparent to participants by altering the proportions of relative topography and relative route lengths, and to ensure that when variable length routes exist, participants would indeed select shorter routes. Filler images were sourced identically as experimental images but did not conform to the topographical guidelines; instead, they depicted mixed proportions of flat and mountainous regions. Further, filler images always depicted two routes characterized by an approximately 40% difference in path length, resulting in one perceptibly shorter route option. Fillers were designed to conform to two factors: Route Direction (North to South, South to North, East to West, and West to East), and Route Length (Left long/Right short, Right long/Left short).

## Procedures

Participants completed an approximately 1-hour session. After providing written consent, they completed a series of four questionnaires: the Santa Barbara Sense of Direction scale [26], the Measure of Environmental Spatial Strategies [27], the Need for Closure Scale [28], and the 45-item Big Five Personality Inventory [29]. The first two measures were selected to measure spatial skills and strategies, the third to measure decision style, and the final to measure relatively domain-general personality traits.

Participants were then presented with all 368 images, one at a time in random order, presented in the center of a 22” computer screen running at 1920×1200 resolution. A compass rose was depicted in the lower right corner (Fig 1). Participants were simply instructed to *“Select the best route from point A to point B*, *by clicking on the route*.*”* They had unlimited time to respond to each image, and responses were made by mouse clicking on their chosen route; on average, participants took 4.4 seconds (*SD* = .39) to respond to each image. After responding to all 368 images, they were asked to report (in a text box) any strategies they may have used when selecting routes. Participants were then debriefed and compensated for their time.

## Results

To ensure that participants intended to select the route in question for all included trials, trials were excluded for clicks not falling within 50 pixels of either route option; removed data constituted 2.9% of all data. Data are included in (S1 Dataset).

### Filler Trials

For filler trials, we examined the proportion of shorter routes selected. For the following analyses, *t* statistics are derived from one-sample t-tests comparing to 0.50 (chance); effect sizes provided using Cohen’s *d*.

On east/west trials, participants selected shorter routes on the vast majority of trials (*M* = .89, *SD* = .12, *t*(99) = 30.59, *p* <.001, *d* = 6.15). The same pattern was found on north/south trials (*M* = .85, *SD* = .17, *t*(99) = 20.49, *p* <.001, *d* = 4.12). Together, these data demonstrate that participants understood the task and were generally attempting to select length-optimal routes.

### Experimental Trials

To test for possible interactions among our factors, we compared route selections conforming to the critical scenarios: selected path was flat versus mountainous, straight versus winding, and south- versus north-going. Selection frequencies were calculated for east/west and diagonal trials where initial segment straightness (straight versus winding), cardinal direction (south versus north), and relative topography (flat versus mountainous) were conflicted in a factorial manner. For analysis, we measured whether the selection of a north or south route was influenced by variation in initial segment straightness and relative topography; for parsimony we report on southern route selection given that the summed proportion of north and south selection always equals 1. Note that across east/west and diagonal trials, the overall proportion of southern routes selected approximated chance (*M* = .49, *SD* = .07, *t*(99) = 1.38, *p* = .17, *d* = .28).

**Fig 2:**
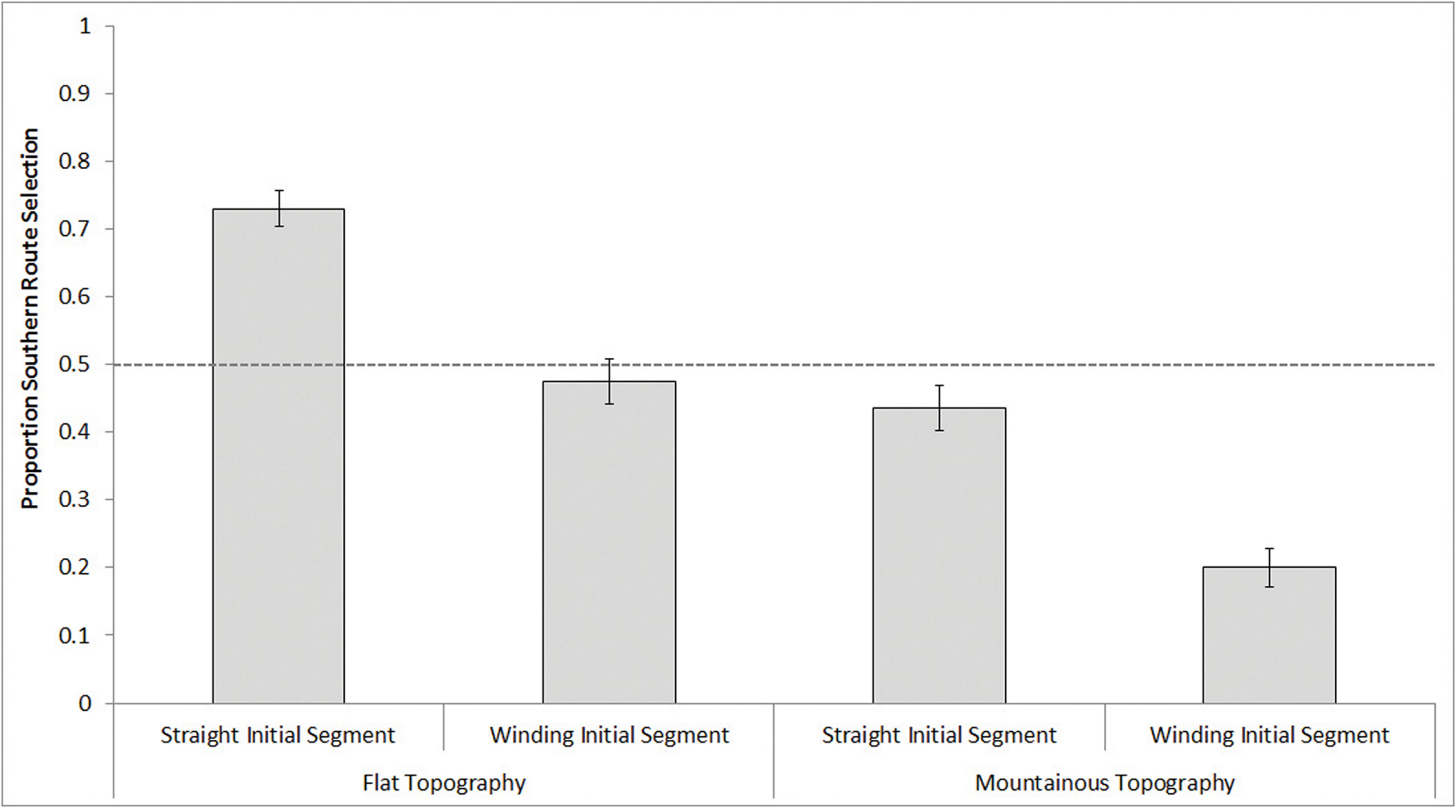
During east/west trials, proportion southern route selection as a function of the southern route’s Initial Segment Straightness and Relative Topography. A value of 0.50 reflects equal proportion selection of south and north routes.

For east/west trials, a 2 (Initial Segment: straight, winding) × 2 (Relative Topography: flat, mountainous) repeated-measures ANOVA revealed main effects of Initial Segment, *F*(1, 99) = 41.55, *p* <.001, *d* = .69, and Topography, *F*(1, 99) = 47.49, *p* <.001, *d* = .79, but no interaction, *F*(1, 99) = .11, *p* = .74. Overall, participants showed a preference for relatively straight initial segments (*M* = .58, *SD* = .22, *t*(99) = 3.22, *p* = .002, *d* = .32), and relatively flat topography (*M* = .60, *SD* = .28, *t*(99) = 3.6, *p* <.001, *d* = .36). These effects are depicted in Fig 2. These patterns were additive; the highest overall chance (73%) of selecting a southern route was in conditions of a straight initial segment and flat topography, and the lowest overall chance (20%) was in conditions of a winding initial segment and mountainous topography.

**Fig 3:**
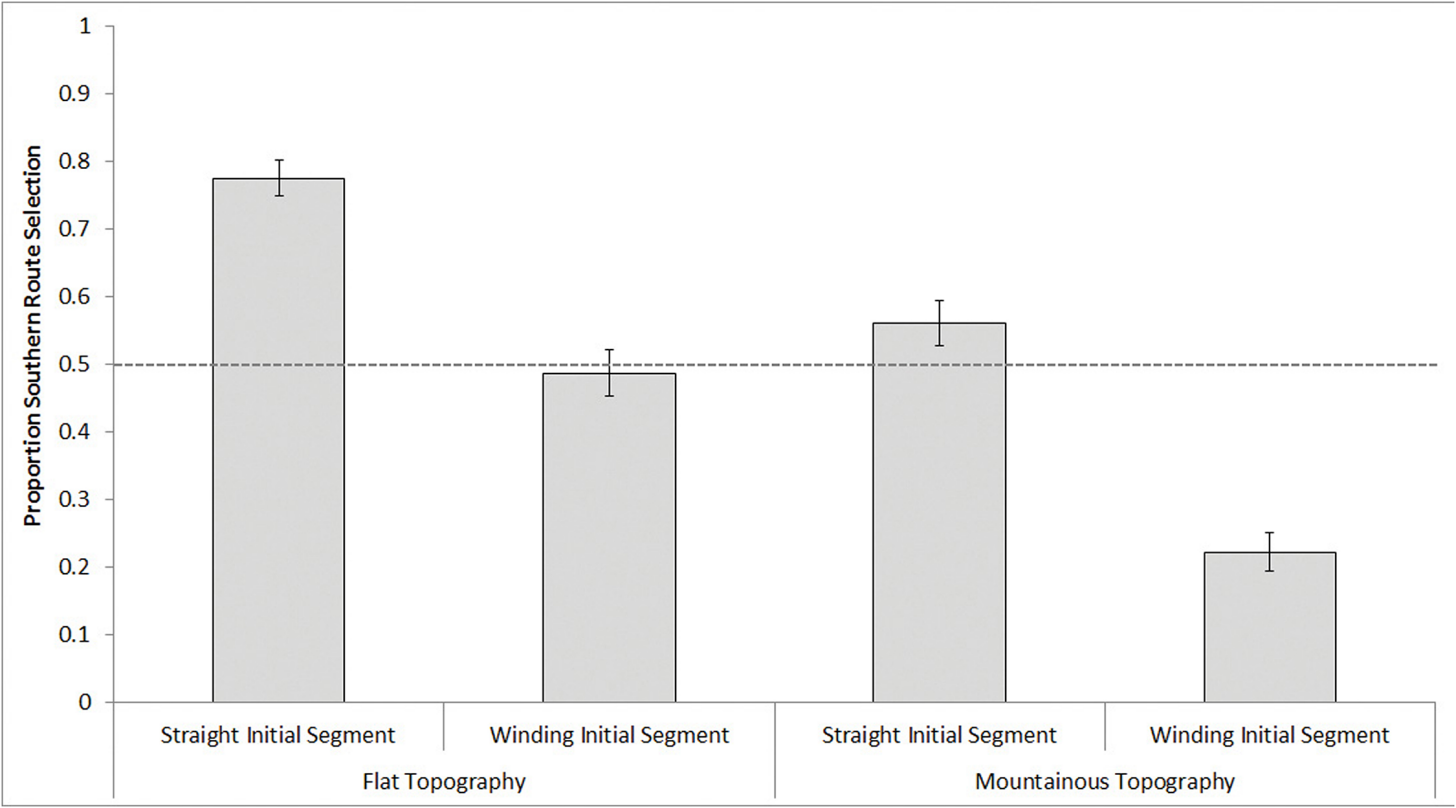
During diagonal trials, proportion southern route selection as a function of the southern route’s Initial Segment Straightness and Relative Topography. A value of 0.50 reflects equal proportion selection of south and north routes.

For diagonal trials, a 2 (Initial Segment: straight, winding) × 2 (Relative Topography: flat, mountainous) repeated-measures ANOVA revealed main effects of Initial Segment, *F*(1, 99) = 89.26, *p* <.001, *d* = .88, and Topography, *F*(1, 99) = 35.5, *p* <.001, *d* = .79, but no interaction, *F*(1, 99) = 1.31, *p* = .26. Overall, participants showed a preference for straight initial segments (*M* = .67, *SD* = .19, *t*(99) = 8.4, *p* <.001, *d* = .84), and relatively flat topography (*M* = .63, *SD* = .24, *t*(99) = 5.43, *p* <.001, *d* = .54). These effects are depicted in Fig 3. Again, these patterns were additive: the highest overall chance (77.4%) of selecting a southern route was when it had a straight initial segment and traversed flat topography, and the lowest overall chance (22.3%) was when it had a winding initial segment and mountainous topography. Note that on diagonal trials, a small but significant southern preference emerged (*M* = .52, *SD* = .07, *t*(99) = 2.29, *p* <.05, *d* = .46).

Thus, we support our first three hypotheses, finding evidence that route selection is independently influenced by cardinal direction, initial segment straightness, and relative topography, and also shows an additive influence of initial segment straightness and relative topography. The effect of cardinal direction, however, was restricted to diagonal trials; we return to this finding in the discussion section.

#### Individual differences

**Fig 4:**
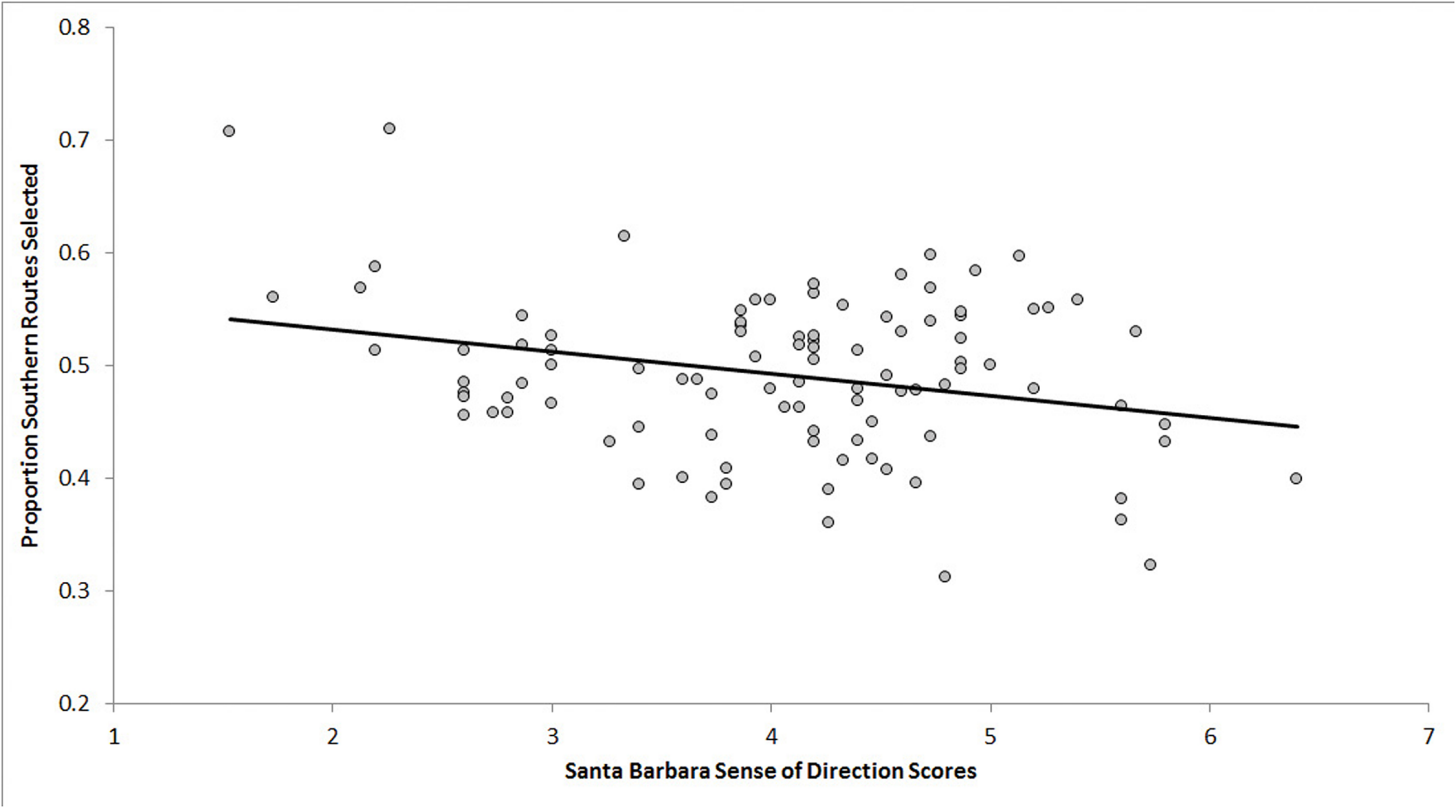
Scatterplot depicting linear relationship between self-rated sense of direction (*x* axis) and proportion southern routes selected (*y* axis).

Our final hypothesis proposed that individual differences in decision making style, spatial preference, and personality traits might influence whether participants consider initial segment straightness, cardinal direction, and/or topography during route selection. To examine this possibility, we conducted three multiple regressions, each including 10 predictors: need for closure composite scores (*M* = 3.61, *SD* = .69), Santa Barbara Sense of Direction scores (SBSOD; *M* = 4.04, *SD* = .99), the 5 scores (extraversion, agreeableness, conscientiousness, neuroticism, and openness) from the Big 5 personality inventory (*M’s/SD’s* = 27.2/2.9, 30.1/2.8, 32.4/3.1, 26.4/2.9, and 37.1/4.1, respectively), and the three scores (egocentric, survey, cardinal direction focus) from the FRS preference questionnaire (*M’s/SD’s* = 4.1/1.2, 3.6/1.4, 2.2/1.4, respectively). The first regression asked whether these 10 measures predicted the extent to which topography was considered during route selection (derived from proportion selection of relatively flat routes during east/west and diagonal trials). The overall model was non-significant, *F*(10, 99) = 0.81, *p* = .61, *R* ^2^ = .08, and showed no significant value of individual predictors (*p*_min_ = .06). The second regression asked whether the 10 measures predicted the extent to which initial segment straightness was considered during route selection (derived from proportion selection of straight versus winding initial segments during east/west and diagonal trials), and was non-significant, *F*(10, 99) = 1.4, *p* = .19, *R* ^2^ = .14; one individual predictor reached significance, with higher levels of extraversion predicting reduced preference for initially straight path segments, *t*(99) = 2.28, *p* <.05, β = -.007 (other *p*_*min*_ = .08). Finally, the third regression asked whether these 10 measures predicted the extent to which cardinal direction (north versus south) was considered during route selection (derived from proportion selection of southern routes during east/west and diagonal trails). The overall model was significant, *F*(10, 99) = 1.95, *p* <.05, *R* ^2^ = .18; and two individual predictors showed significance. First, higher levels of extraversion predicted lower preference for southern routes, *t*(99) = 2.56, *p* <.05, β = -.006. Second, higher sense of direction (SBSOD) scores predicted lower preference for southern routes, *t*(99) = 3.2, *p* <.01, β = -.03 (Fig 4). Extraversion and SBSOD were not correlated (*p* = .81).

Thus, regression analyses suggest some limited value of one personality trait (extraversion) and the SBSOD, but not other measures of personality or spatial preferences, in predicting route selection. Specifically, individuals with higher extraversion tended to show lower preference for flat topography or south-going routes, and individuals with higher sense of direction showed a lower southern route preference.

## Follow-Up Analyses

**Fig 5:**
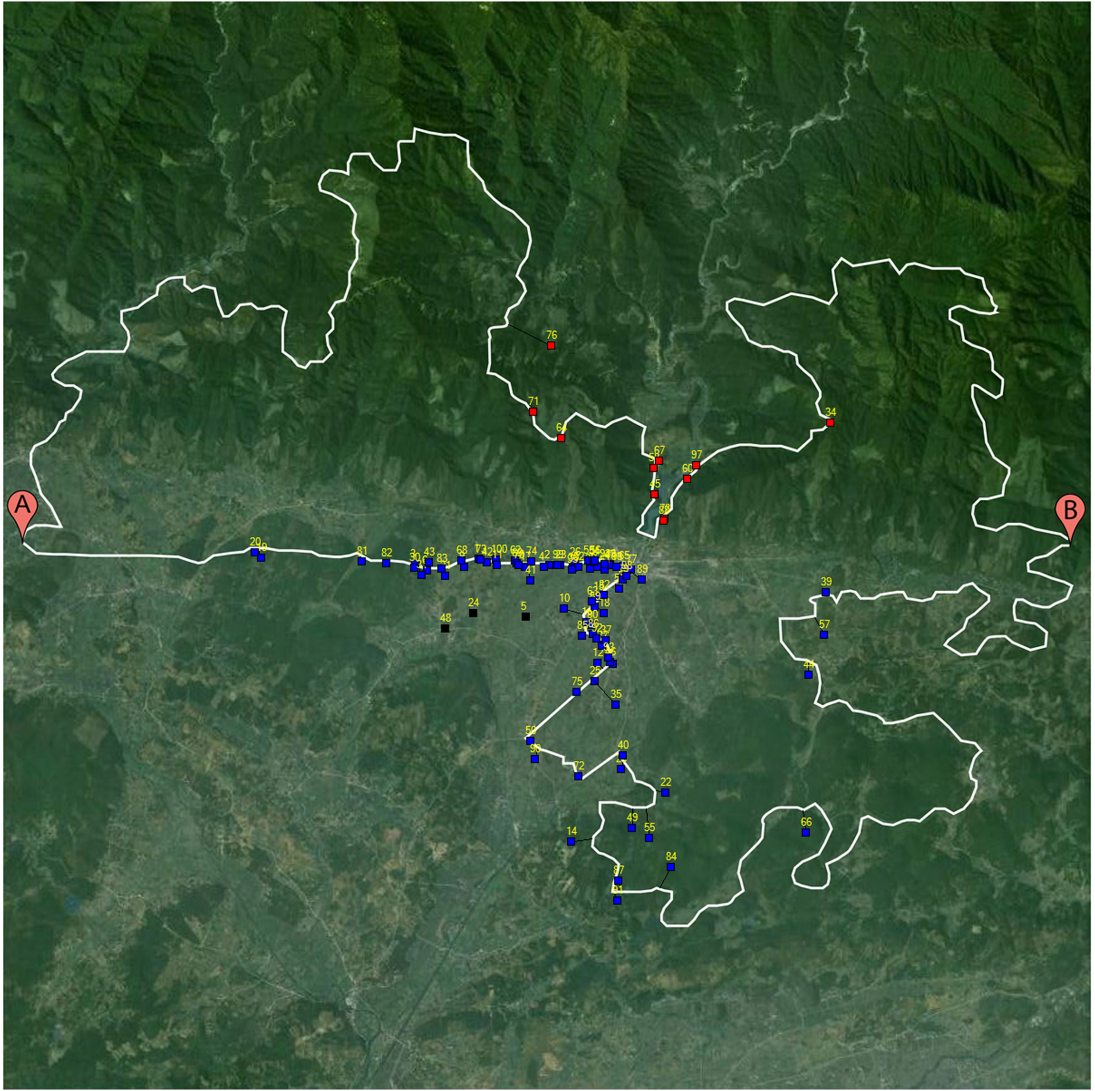
An example trial demonstrating a qualitatively dense click distribution toward the end of an initially straight segment. Blue and red markers indicate a click scored as southern and northern route selections, respectively. Black markers indicate a click outside of the 50-px scoring threshold.

Our procedure afforded precise understandings of mouse click coordinates during route selection. As a preliminary step in our analyses, we plotted participants’ click locations onto each of the 368 images. Visual inspection of these plots revealed a qualitative finding of interest. As depicted in Fig 5, we noticed that during trials with an initially straight segment, participants show denser click distributions toward the first turn of the initial segment. In contrast, click locations tend to be distributed across the length of the route in conditions without an initially straight segment. Together, these patterns suggest that when an initially straight segment is available, participants focus attention on the initial segment of the route; when no initially straight segment is available participants tend to consider the entire route, as evidenced by relatively distributed click locations. Continuing data collection should assess whether click locations (and perhaps mouse dynamics; [30,31] might prove valuable in revealing information about decision criteria employed during route selection.

Because our origins and destinations were reversed across image versions, we also examined whether the direction of routes (e.g., going east to west, or west to east) influenced route selection patterns. There was no reliable evidence that the direction of routes influenced the application of any of our three strategies.

Though our cardinal direction analyses tended to be focused on east/west and diagonal routes, we also examined north/south routes for any tendency to select routes that went generally west versus east. On these trials, participants showed a moderate tendency to select western rather than eastern routes (*M* = .53, *SD* = .09; *t*(99) = 3.43, *p* <.001, *d* = .69). In past samples, we have found unreliable evidence for both western and eastern route preferences, tending to range from 0–5% [4,9]. This study is the first to find any east or west preference reaching significance.

Finally, we also analyzed response times for potential differences as a function of route selection. When participants selected southern versus northern routes, they showed highly similar response times (*M*_*south*_ = 4654.2ms, *M*_*north*_ = 4655.2ms). Participants took longer to respond when they selected a winding route (*M* = 4651.4) versus straight route (*M* = 4431.6), *t*(99) = 4.33, *p* <.001, *d* = .87. Participants also took longer when they selected a route that traversed mountainous (*M* = 4788.6) versus relatively flat (*M* = 4170.9) regions, *t*(99) = 3.9, *p* <.001, *d* = .78.

## Discussion

The present study was aimed at understanding the relative influence of route and topographical image features used in guiding route selection: the initial straightness of a route segment, the relative topography traversed by routes, and the extent to which the route heads generally north or south toward its destination. In general, we find strong evidence that participants rely upon initial segment straightness and relative topography when selecting routes. We also find evidence for a southern route preference, but this preference only emerges when routes connected points diagonally positioned on the image. Finally, we find some limited evidence that individual differences in self-reported sense of direction predict southern route preferences. We discuss each of these findings in turn, with implications for theory and continuing research.

### Southern Route Preference & Initial Segment Strategy

Some recent theory [13] suggests that route planners may focus on perceptible features of early route segments, such as their straightness [8] or initial cardinal direction [9]. In doing so, route planners theoretically employ cognitively efficient rules of thumb [15]. Evidence for initial segment reliance and southern route preferences strongly support these theoretical positions. The present data suggest that initial path segment straightness is a dominant path feature for selecting between routes, a feature that can be ascertained by focusing close to the origin. We propose that the southern preference is similarly related to a reliance on initial path segments rather than the relatively global properties of an entire route. Indeed the extent to which an initial segment is oriented directly south seems to predict route selection. For instance, our diagonal routes, specifically those traveling from northwest to southeast, or northeast to southwest, have initial southern segments that tend to orient directly south. Follow-up analyses showed that in these specific conditions participants showed a more pronounced southern route preference (57.4%, *t*(99) = 3.09, *p* <.01, *d* = .62); as would be expected, they did not show this preference when the route traveled in the opposite direction (50%; *t*(99) = 0.02, *p* = .98, *d* <.01).

However, when a focus on early route features alone fails to effectively distinguish route options, participants likely consider relatively global features of a path such as continuously averaging the angular difference between path positions and the goal to select the option [17], or considering large-scale topographical variation [12]. In the present data, this is evidenced by the relatively lengthy response times when individuals ultimately selected a winding rather than straight initial path segment. Indeed, considering the entirety of a route is a rather time-consuming process. Navigators may consider this increased planning time and whether it may negate the advantage of selecting an optimal route. In other words, the time spent determining geometrically ideal routes may exceed the additional time spent navigating a relatively suboptimal route [7]. Thus, a focus on the initial segment’s straightness and cardinal direction may reflect effort toward cognitive economy. Straightness and heading in the general direction of a destination likely correlate with a route’s directness [6], and heading south is implicitly associated with minimizing physical effort (i.e., south is downhill; [32]). Together, these two characteristics of an initial path segment strongly predict path selection through relatively naturalistic landscapes.

### Topography

The present study also uniquely explored the influence of topography, a relatively large-scale environmental feature that may guide route selection particularly when route planners do not solely rely upon initial path features. Golledge [12] suggested that relative topography might influence route planning, though with the exception of modeling work [11], to our knowledge no research has examined the extent of topography’s influence on route selection. With the large-scale environments used in the present work, we find reliable evidence that participants avoid relatively elevated topographies when selecting routes. Rationale for considering topography during route planning seems obvious. Indeed energy expenditure related to traversing mountainous topography is quite high, particularly during uphill regions, and models reliably define hiking functions strongly relating terrain slope to time and energy expenditure [11,19,33]. Research also shows that humans reliably attend to and often overestimate slope, and this can influence perceived affordances (i.e., *What is the cost associated with taking a particular route*?) during locomotion [34]. Thus, it is not particularly surprising that participants would rely on relative topography during route selection, and this effect is likely due to implications for time and/or energy expenditure. Of course, it is unknown whether relative topography will differentially influence route selection when time costs are minimized (i.e., for tourism), or energy expenditure is minimized (i.e., when driving versus hiking).

### Relative & Additive Influences

The present study provided a unique opportunity to examine the relative, and potentially interactive, influence of multiple wayfinding strategies. The three strategies examined differed markedly in their relative influence, with the strongest overall influence being relative topography, followed by initial segment straightness, and then cardinal direction. Topography appears to show the greatest influence in terms of both extent and reliability. Also, when participants select a route traversing a relatively flat region, they do so quickly, suggesting that it provides a relatively rapid solution for the decision making process, perhaps due to its direct relevance in calculating cost functions [11].

We also found that the influence of these three strategies was additive in nature. Indeed the highest likelihood of selecting a route, for instance during diagonal trials, was when the route went generally south, had a straight initial segment, and traversed relatively flat topography. The lowest likelihood was when all three of these factors were reversed. There were no clear interactions among these factors, as all three seemed to play a role in the presence or absence of other route features.

### Individual Differences

We found evidence that only two of our predictors, extraversion scores on the Big Five Personality Inventory, and the Santa Barbara Sense of Direction (SBSOD), predicted strategy application. Specifically, participants with higher extraversion scores showed a lower preference for routes heading south or traversing relatively flat topography. Also, participants with a lower self-reported sense of direction showed a greater tendency to select southern rather than northern routes.

In general, extraversion describes individual propensities to be talkative, assertive, and energetic, and show tendencies toward physical activity, risky behavior, and excitement-seeking [29,35]. It could be the case that individuals with high extraversion may seek higher physical activity and adventure by pursuing relatively difficult terrain. In the present study this may manifest by showing a reduced tendency to select routes traversing relatively flat topography. It may also be the case that high extraversion and social assertiveness may be related to activating associations between social dominance and higher locations along the vertical axis [36], to include locations to the north rather than south [31]. The SBSOD measures the general ability to orient oneself in large-scale environments, and is related to several environmental-scale tasks such as pointing to familiar landmarks, learning new layouts, and blindfolded updating [26]. The present data suggest that extended experience in real-world environments, and fluidity in learning and negotiating such environments, might transfer to abstracted map-based route planning tasks. It could be the case that individuals with higher sense of direction are better able to understand the nature and experience of varied allocentric heading, potentially showing lower implicit associations of coordinate space with the vertical axis (i.e., north-up, south-down). It could also be the case that individuals with higher sense of direction scores tend to have more extended experience with maps and route planning, and thus are more knowledgeable about the fallibility of such associations.

## Conclusions

The present study helps inform theoretical models of route selection, and aids modeling efforts that attempt to predict route selection across domains. Considering the relative influence of multiple spatial strategies may help inform applications such as locating isolated personnel, recovering the lost, and traffic management [23–25]. Indeed the present data will complement extant data in populating predictive models for locating and recovering isolated personnel. Identifying the full range and relative influence of spatial strategies on human behavior is critical to fully understanding and predicting human navigation. While we have motivated our work by considering extant research from both navigation and map study, we also note that further research is needed to better understand whether results found with maps or satellite images may generalize to virtual or real-world navigation behavior.

In sum, the present study was aimed at understanding the relative influence, and potential interactivity, of three perceptible geospatial features in guiding route selection. We further explored whether spatial or domain-general individual differences in skills, preferences, and personality traits might predict any such effects. Data demonstrate reliable influences of all three features, but show particular emphasis on the straightness of initial path segments and relative topography in guiding route selection. Participants with lower extraversion or self-rated sense of direction also tended to show higher reliance on the cardinal direction of routes, showing an increased tendency to select southern rather than northern routes. Together, results demonstrate that navigators consider multiple geospatial factors when selecting routes, but these factors differ in relative influence and vary in their application across individuals.

## Supporting Information

S1 Dataset Route selection data for east-west, north-south, diagonal, and filler routes. For east-west routes, scores of 1, 0, -1 indicate north, none, and south selection biases, respectively. For north-south routes, scores of 1, 0, -1 indicate east, none, and west selection biases, respectively. For diagonal routes, scores of 1, 0, -1 indicate north (NW or NE), noe, and south (SW or SE) selection biases, respectively. For filler routes, scores of 1, 0, -1 indicate north, none, and south selection biases, respectively.
(XLSX)
